# Gelsemium in Convulsions*This is the orthography of the late edition of the Pharmacopœia, in which the editors seem to have followed Prof. Gray, in his Botany, in an innovation which has no warrant but the caprice of the author. I adopt it, because it is almost a necessity to have a uniform usage, and it is not convenient to establish any other than that of the Pharmacopœia.

**Published:** 1863-09

**Authors:** Henry Wing

**Affiliations:** Professor of General Pathology and Public Hygiene in the Chicago Medical College


ARTICLE XXIV.

### GELSEMIUM* IN CONVULSIONS.

By Dr. HENRY WING, Professor of General Pathology and Public Hygiene in the Chicago Medical College.

Some years ago, in using gelsemium, I found that the mark of its distinct impression was, generally, a drooping of the upper eyelids. In some cases, which were, generally, those in which the drug had been pushed farther, dimness of vision was complained of, and sometimes the patient would say he could not see. As the optic nerve has its origin somewhat remote from the one most commonly affected, and as the muscles which direct the eyeball are supplied in common with the *levator palpebrarum* by branches of the *motor oculi*, it suggested that the defect of vision might be owing to unsteadiness and want of coördination of the eyeballs. Accordingly, in a number of cases, I asked the patient to cover one eye with the fingers and look with the other. In this way, they said, that they could see. I do not remember any case where this trial failed of the same result, but the number of instances where I pushed the remedy to that extent was not large.

In one case which came to my knowledge, but I did not see, the tongue was so far paralyzed as to render articulation impossible, though the voice was not impaired. In another, where I prescribed the medicine, but did not see the patient under its influence, he reported that he lost all control of himself, and lay upon the bed wholly unable to rise or lift his hand. The article employed in this case was gelsemine, (which I do not regard as of uniform strength,) and the dose was two grains, having been increased from half a grain, at periods of an hour, until that amount was reached, and the effects above-mentioned.

* This is the orthography of the late edition of the Pharmacopœia, in which the editors seem to have followed Prof. Gray, in his Botany, in an innovation which has no warrant but the caprice of the author. I adopt it, because it is almost a necessity to have a uniform usage, and it is not convenient to establish any other than that of the Pharmacopœia.

It may be worth while to state, that the case just mentioned was one of osteosarcoma of the upper maxillary bone, involving the nose on one side, and the infraorbital firamen. The medicine was given as an experiment, to allay the agonizing pain. The patient seemed to regard it as successful in that respect, but he was so much alarmed that he was unwilling to repeat the experience.

Having found, by the above cases, that gelsemium suspended the action of some of the voluntary muscles, and was capable, in some cases, of so affecting them all, I thought this property might be made useful in cases where the muscles act inordinately and beyond the control of the will, as in tetanus, and some forms of convulsions, where the muscular action is both an evil and a source of mischief. Accordingly, I commenced to employ it in such cases, as I had opportunity. Tetanus has never occurred to me to treat; but in puerperal and other convulsions I repeatedly used it with apparent advantage. In February, 1857, I employed it in a case of cerebro-spinal meningitis, restrainig, apparently, the muscular contractions and, perhaps, contributing to the cure which was effected under circumstances not encouraging. In all these cases, however, there was room for doubt as to whether the medicine really had the effect supposed. It was the common experience of medical uncertainty, with an affirmative presumption. Recently, I had an opportunity of trying its effects under circumstances almost equivalent to a demonstrative experiment.

I had a patient subject to paroxysms of convulsions, which could be positively recognized in their approaches, and, when once distinctly initiated, they never failed to culminate in well-marked opisthotonos, lasting a number of hours. The case had been in the care of a very accomplished physician in St. Louis, who had skilfully employed upon it most, if not all, the recognized resources of our art, with but partial and varying success. At first, I endeavored to avail myself of the results of his experience in the case, and to that end I pushed to a full impression quite a number of articles, with a view to arrest the paroxysms when they were evidently coming on. I may specify, in this connexion, morphine, ether, chloroform, both alone and in combination, and the two volatile articles, both by inhalation and ingestion,—cannabis Indica, valerian, assafœtida, and nasueants. Some of the more powerful of these would exercise a certain influence over the symptoms, but they would still continue very near their natural term. After a severe paroxysm had exhausted itself, sleep generally followed of a very sound character.

It may be further stated, that the case was one of enlargement and induration of the uterus, associated with great hyperæsthesia of that organ. There was also some tenderness of the fourth dorsal vertebra; and ice passed over that part gave a sensation of heat. To avoid a lengthened detail, I will merely state, that I regarded the spinal symptoms as secondary in their origin and not owing to organic change in the spine itself.

After having fully tested the means previously mentioned, I determined to make a vigorous effort with gelsemium and push it to the verge of excess. An opportunity presented itself August 3d. After ten in the evening, the usual symptoms of a paroxysm began to manifest themselves:—First, there was pain in the womb, then in the back and head, accompanied by constant activity of the fingers, twisting up edges of the bed-clothes, drumming, etc., which had the appearance of being voluntary; but whilst the will could direct the movements, it could not stop them; frequently the arms, and at other times the lower extremities, would be tossed about. When the symptoms were a little more advanced, there would occur a sudden start of all the muscles, as if a flash of electricity had been sent through the whole body, and the head and legs began to be uncontrollably bent backwards.

After these manifestations had reached the degree just described, having been over an hour in progress, and it being quite certain that, without more successful treatment than had yet been tried, the violent convulsions would cone on and consume the rest of the night, I commenced the administration of gelsemium. The article used was a fluid extract, made by J. S. Merrill, of St. Louis, and stated by him to be about four times the strength of the common tincture. I gave of this thirty drops. In three-quarters of an hour there was no abatement of the symptoms, neither had they decidedly advanced. There was a little heaviness of the upper eyelids. I repeated the dose. In ten or fifteen minutes, the patient began to be quiet, to keep her eyes closed, and to sleep. In half an hour, she said, that she could not see, and she began to sleep very soundly, as she did, generally, when the paroxysm of convulsions had spent itself. The sleep became so heavy that I thought it might be prudent to counteract it with coffee. Coffee was brought, and I asked her to cover one eye and endeavor to see with the other. She took a little of the coffee, covered the eye as requested, but could not keep the other open long enough to ascertain whether she could see or not. Afterward, she said, in regard to this, that her eyes seemed crossed, and dancing in her head. She remained intensely drowsy (if such an expression may be allowed,) all night. There seemed to be a struggle between the two influences operating upon the system. At times, she would throw herself across the bed, as she was accustomed to do in the beginnings of the paroxysms, and would pass her hand in an excited manner over the hypogastric region, then relapse into sleep again, all the time having the eyes closed. When interrogated on the subject, she said, that the uterine pain continued as before.

August 10th, one week after this, I had an opportunity of repeating the experiment on the same patient. Twitchings of the fingers and a sort of subsultus began about 10 o'clock. This extended to the limbs, causing the patient to throw herself in a restless manner from one side of the bed to the other from time to time. At intervals, sudden startings of the whole body would occur like electric shocks, followed by bending the head and limbs somewhat firmly backward. The patient made a most determined effort to control the movements by strength of will, but they increased in force. At 12 o'clock, I found the symptoms as above. Thinking that in the former case I had used more of the gelsemium than necessary to control the symptoms, I endeavored, by small and repeated doses after one full one, to ascertain the amount necessary. I ordered thirty-five drops to be administered, and ten more to be given every ten minutes. The direction was not strictly carried out, but it was done as follows:—

| | | | |
|---|---|---|---|
| 12 P.M., | ........................................ | 35 | drops. |
| 12.20 P.M., | no effect,...................... | 10 | do. |
| 12.30 do. | do. ...................... | 10 | do. |
| 12.40 do. | do. ...................... | 10 | do. |
| 12.55 do. | slight heaviness of eyelids, | 10 | do. |
| 1.5 do. | no increase of effects,.... | 10 | do. |

Finding this state of the case, I ordered the doses to be doubled. Accordingly, at 1.15 twenty drops were administered, and in ten minutes more the heaviness of the eyelids was becoming almost uncontrollable and the muscles, generally, becoming quiet. No more medicine was given. The eyes could be seen to be tremulously moving under the imperfectly closed lids. Sleep came on, but at intervals, for a short time, the patient would seem to arouse and turn over or throw herself into a new attitude with a sort of struggle, as if trying to awake; but the sleep became more continuous and extended into the morning, contrary to the habit of the patient, who is a poor sleeper. In the morning she rose up suddenly, and, looking at the clock, said, "Why! it is 8 o'clock!"

I do not wish to be understood as considering this as evidence of a soporific effect of the gelsemium. I have never found it to act in that way. Before commencing the administration in the case above, I made several attempts to ascertain the character of the pulse, which were unsuccessful on account of the constant action of the muscles. When it became practicable to examine the pulse, under the influence of the medicine, it was found to be equable, only moderately full, and eighty beats to a minute. So far as could be judged, it was not materially changed. On the following day, though she promptly read the hour on the clock at eight, she was unable to read a letter until some time in the afternoon, either with both eyes or one.

August 12th.—The patient being out of my reach, and feeling the approaches of a paroxysm, herself asked for the medicine to be given, according to my directions for such an emergency. Forty drops were accordingly given at half-past seven, and twenty drops more given every half hour until four doses were given. She then said, she thought with ten drops more she would sleep, as she did, resting well until morning, but not oversleeping her usual hour, as in the former case, where the paroxysm was formed before being interrupted.

August 15th.—The threatenings of a paroxysm were distinct. Forty drops were administered, and in half an hour thirty more, with the effect of preventing the paroxysm as before, but the patient was entirely unable to sit up for the remainder of the afternoon.

If subsequent observation in other cases shall accord with the experience related in this article, we have in gelsemium a medicine almost exactly antagonistic in its action to strychnia,—capable of restraining involuntary muscular action, as that drug excites it, and having a wide range of practical applications.

In tetanus, great benefit might be expected from such a remedy. In puerperal convulsions, the removal of the pathological cause often requires time, during which, the recurrence of the paroxysms is a cause of anxiety. Any means which would prevent these would be valuable. In poisoning by strychnia, when not speedily fatal, but a tetanic state is induced, of suffering and danger, there would be time to get the counteracting effect of gelsemium. Many other uses will suggest themselves to the practitioner.

How much is required to produce the effects considered? I have not the means of giving a definite answer to this question. I have stated the amount I have used, of the article I had. If correctly informed of its strength, the doses were about three times those generally administered for other purposes. In the case where I first produced general paralysis, it will be remembered that the dose was two grains of gelsemine following one grain and a-half at a period of one hour.

Is it safe? I think that when carefully proceeded with the drooping of the eyelid is sure to be in advance of the profound impression, and something short of it. It does not seem to be ever cumulative. Is it followed by any bad consequences? So far as my observations have allowed me to judge, the profound impression upon the voluntary muscles has not been accompanied by any effect upon the involuntary muscles or the functions of organic life. In all the cases where I have seen impairment of vision, no trace of it has remained, nor any other disagreeable effect.

Of course, impressions so profound upon the nervous system will never be resorted to by the intelligent practitioner, either recklessly or without due consideration and care. What might be the effect of pushing so powerful a drug farther than to the extent of producing general paralysis, others can conjecture as well as I. Without some unfortunate accident, it may be hoped it may never be settled by experience, unless upon some of the lower animals.

